# An Observational Study on Clinical Insights and Outcome Prediction of Lupus Enteritis in Indian Systemic Lupus Erythematosus Patients

**DOI:** 10.31138/mjr.300924.ioe

**Published:** 2025-06-30

**Authors:** Sowmya Kotha, Ritasman Baisya, Keerthi Vardhan Yerram, Arjun Kumar Ramavath, G S R Murthy, Phani Kumar Devarasetti, Liza Rajasekhar

**Affiliations:** 1 Clinical Immunology and Rheumatology, Nizam’s Institute of Medical Sciences, Hyderabad, India;; 2 Research Professor, Indian Institute of Technology, Hyderabad, India

**Keywords:** lupus enteritis, recurrent, prediction, mortality

## Abstract

**Background:**

Lupus enteritis (LE) is the most common serious gastrointestinal manifestation of SLE. Indian literature on LE is limited, while most of the data comes from China and Western series with recent efforts on developing prediction models for its occurrence & recurrence**.**

**Objectives:**

The objectives of the study were to analyse clinical and laboratory parameters of LE, to compare recurrent versus non-recurrent LE and to identify predictors of its recurrence and mortality.

**Method:**

Patients with LE who were admitted to the Rheumatology ward from 2018–2022 were considered cases. For cases, symptoms, abdominal CT findings, and outcome (recurrence or death) were recorded. Logistic regression was used for the prediction of recurrence and mortality.

**Result:**

Among 48 LE patients, 45 were female. The mean (± SD) age of the first enteritis episode was 27.04± 8.92 years. The most frequent extra-gastrointestinal manifestations were nephritis and arthritis (54% of cases). Mean disease duration, lupus nephritis, arthritis, and mean SLEDAI were higher in non-LE patients. Thirteen (27.1%) patients had recurrent LE & hydroureteronephrosis was more prevalent in them (p = 0.002). The logistic regression model using dsDNA complements and albumin failed to predict recurrence. Multiple repetitions of the confusion matrix for the mortality prediction model yielded variable ρ scores, suggesting insignificant accuracy prediction.

**Conclusion:**

Colon and bladder involvement is more frequent in recurrent LE. Anti-dsDNA antibodies, complement, disease activity index, and serum albumin failed to predict recurrence and mortality in our cohort.

## INTRODUCTION

Systemic lupus erythematosus (SLE), a multisystem autoimmune disorder, can exhibit varied gastrointestinal manifestations, with lupus enteritis (LE) being the most common serious entity.^1^ It is primarily caused by intestinal small vessel vasculitis. However, circulating antiphospholipid antibodies may also contribute to its pathogenesis by intestinal vessel thrombosis. The prevalence of LE is reported to be 0.2–6% in Western literature^2,3^ and 3–6% in Asia.4 Early recognition and initiation of treatment are crucial as LE can potentially lead to a fatal situation. Most of the available data on LE comes from China, Korea and Western series, with recent efforts concentrating on developing prediction models for its occurrence and recurrence. However, the literature on clinical characteristics and treatment outcomes of LE in the Indian SLE population is limited to case reports and case series. Hence, there was a need for original research on LE focusing on its long-term prognosis in India. Our study aimed to collect comprehensive data on LE patients from a single centre in India and identify the factors associated with its recurrence and mortality. This study also employed prediction analysis to gain insights into the prognosis of LE.

## OBJECTIVES

The primary objective of the study was to analyse the clinical and laboratory parameters of LE and compare them with those of non-LE patients. The secondary objectives included comparing recurrent versus non-recurrent LE and identifying predictors of its recurrence. We also attempted to develop a prediction model for mortality in LE.

## METHOD

### Study design

This was a hospital-based observational study. Part of the data was retrieved from the Lupus Registry in our hospital from 2015 to 2022 and followed up by either a telephone consultation or an outpatient visit with ethics approval number EC/1181/2009 obtained from NIMS institutional ethics committee. Another subset of patients was recruited directly during admission in 2022 (at the time of the study), with subsequent follow-up.

### Study participants

All participants met the classification criteria for SLE as defined in either the 1997 classification of SLE by the American College of Rheumatology (ACR)^5^ or the 2012 classification by the Systemic Lupus International Collaborating Clinics (SLICC).^6^ LE was diagnosed based on the presence of three conditions: (i) abdominal symptoms such as abdominal pain, diarrhoea, bloating, nausea or vomiting; (ii) at least one of the following three imaging manifestations: bowel wall thickening, oedema or increased attenuation of the mesenteric fat; and (iii) clinical response with glucocorticoids and immunosuppressant therapy.^7^ We excluded SLE patients with infective causes of enteritis. Written or verbal consent was taken from all patients. The study was conducted as per the declaration of Helsinki. For each lupus enteritis (LE) patient in the consecutive series, two age and sex-matched non-LE patients admitted simultaneously were chosen as controls for comparison.

### Study variables

The following parameters were recorded for each patient in both groups – demographic variables, including age, sex, and disease duration; clinical variables, including SLICC criteria, British Isles Lupus Assessment Group (BILAG) domain-based organ involvement; disease activity variables, including SLE Disease activity index (SLEDAI), dsDNA and complement at the time of admission; treatment modalities including use of corticosteroid, immunosuppressant, and surgical intervention. For all the patients in the LE group, abdominal symptoms (pain, diarrhoea, bloating, nausea, and vomiting), abdominal computer tomography (CT) imaging features (bowel wall thickening, Target sign, increased attenuation of mesenteric fat, hydroureteronephrosis, cystitis and ascites), and drug regimens (maximum dosage of glucocorticoids and use of immunosuppressant, surgical intervention) were recorded.

### Statistical analysis

We used SPSS version 24.0 for our statistical analysis. Discrete variables are presented as frequencies and percentages, which were compared using either the chi-square test or Fisher’s exact test. Continuous variables are reported as mean ± standard deviation (SD) and were compared using the Student’s t-test. To predict recurrence, we applied logistic regression analysis. Additionally, we developed another logistic regression model to predict mortality. The final prediction model included the following variables: age, BILAG-A domain involvement of other major organ systems, serum albumin levels, anti-dsDNA, C3, and C4. We considered differences to be statistically significant when the two-sided p-value was less than 0.05.

## RESULT

### Demography and clinical characteristics

Data from 1500 patients were retrieved from the Lupus Registry and indoor admission records from 2018 to 2022; among them, the total number of LE patients was 48, suggesting its prevalence of approximately 3.2% in our cohort. Among 48 LE patients, 45 were female, with the mean age of the first enteritis episode being 27.04 ± 8.92 years. The mean duration of lupus was 4.8 ± 3.7 years, while the mean duration between the onset of SLE to LE was 2.45 years (± 3.6). The most common manifestation was abdominal pain (89%), followed by diarrhoea (81%) and vomiting (77%) in our cohort. Two patients exhibited features of obstipation, both of whom experienced recurrent episodes and succumbed during their hospital admissions. Ascites, pancreatitis, and hepatopathy were present in 75%, 12.5%, and 23% of cases, respectively. The most frequent extra-gastrointestinal manifestations were nephritis and arthritis, occurring in 54% of cases. These were followed by leukopenia, myositis, malar and discoid rashes, thrombocytopenia, and autoimmune haemolytic anaemia (AIHA). BILAG category A during the episode of LE was the maximum for nephritis (31%). Mean serum albumin, C3, C4, dsDNA and SLEDAI were 2.39 ± 1.0 g/dL, 46.55 ± 32.8 mg/dL, 11.22 ±15.5 mg/dL, 1.87 ±1.62 U, 12.7 ±8.8 respectively. Intravenous methylprednisolone pulse therapy was used in 52% of cases, intravenous cyclophosphamide in 14 cases (29.2%) and injection rituximab in five (10.4 %) cases. Surgical intervention was conducted for two cases (**Table 1**).

**Table 1.** Demographic and clinical characteristics of lupus enteritis patients at admission.

**Parameters**	**Frequency / Mean ± SD**
Age (years) at enteritis presentation (mean ± SD)	27.04 ± 8.92
Females n (%)	45/3
Mean duration between onset of lupus to enteritis (years)	2.45 ± 3.6
Mean duration of SLE	4.8 ± 3.7
Abdominal pain at presentation	43/48 (89.5)
Diarrhoea at presentation	39/48 (81.3)
Vomiting at presentation	37/48 (77.1)
Pancreatitis	6/48 (12.5)
Ascites	36/48 (75)
Hepatopathy	11/48 (22.9)
Malar rash	12/48 (25)
Discoid rash	7/48 (14.6)
Arthritis	26/48 (54.2)
Myositis	15/48 (31.3)
Nephritis	26/48 (54.2)
Serositis	37/48 (77.1)
AIHA	4/48 (8.3)
Leukopenia	21/48 (43.8)
Thrombocytopenia	10/48 (20.8)
Thrombosis	3/48 (6.2)
APL positivity	13/46
Mean serum albumin	2.39 ± 1.0
Mean C3 (mg/dL)	46.55 ± 32.8
Mean C4 (mg/dL)	11.22 ±15.5
Mean DsDNA (U)	1.87 ±1.62
SLEDAI (mean ± SD)	12.7 ± 8.8
BILAG-A	
Mucocutaneous	5 (10.4)
MSK	6 (12.5)
Neurological	7 (14.6)
Cardiopulmonary	6 (12.5)
Renal	15 (31.3)
Haematological	6 (12.5)
Expired	13 (27.1)
Recurrence	13 (27.1)
Intravenous methylprednisolone pulse therapy	25 (52.1)
Cyclophosphamide	14 (29.2)
Rituximab	5 (10.4)
Surgical intervention	2

### Imaging characteristics

Computer tomography of the abdomen was done for all cases. Small and large intestinal involvement was seen in 38 (79%) and 33 (69%) cases, respectively, while both intestines were involved in 25 (52%) cases. Target sign and mesenteric fat stranding were seen in 8 (16%) and 11 (23%) cases, respectively. Hydroureteronephrosis or cystitis was observed in 14 (29%) cases. Endoscopic and colonoscopy abnormalities were seen in 7 (16%) and 12 (27%) cases, respectively (**Table 2**).

**Table 2.** Imaging characteristics in LE patients (CT imaging).

**Imaging characteristics**	**Frequency**
Small intestine involvement	38 (79.2)
Large intestine involvement	33 (68.8)
Both intestines involvement	25 (52.1)
Target sign	8 (16.7)
Hydroureteronephrosis/cystitis	14 (29.2)
Mesenteric fat stranding	11 (22.9)
Upper GI endoscopic abnormality	7/44 (15.9)
Lower GI endoscopic abnormality	12/45 (26.7)

### Comparisons of clinical data in the LE and non-LE groups

**Τ**he comparison of general information (the sex ratio and age at screening) collected from these two groups of patients indicated no significant difference. The LE group’s mean disease duration was higher (p- 0.03). Among clinical parameters, lupus nephritis (p-0.00) and arthritis (p-0.04) were significantly more in the non-LE group, while mucocutaneous, haematological and neurological involvement did not differ statistically. Mean SLEDAI was more in non-LE (p- 0.00), while dsDNA and complement level had no statistically significant difference between the groups (**Table 3**).

**Table 3.** Clinical characteristics between cases vs control.

**Variables**	**LE**	**Non-LE**	**p value**
Mean age	26.6 ± 8.8	26.0 ±8.4	0.73
Sex	3/43	9/75	0.43
Mean disease duration (months)	47.8 ±25.4	37.5 ±25.5	0.03
Mean SLEDAI	13.8 ± 7.4	20.7 ±6.5	0.00
Mean dsDNA (U)	1.9 ±1.6	2.4 ±1.8	0.09
Low complement	38/46	75/84	0.28
Nephritis	26/46	73/84	0.00
Neurological	15/46	20/84	0.28
Arthritis	27/46	64/84	0.04
Mucocutaneous	30/46	65/84	0.13
Haematological	22/46	47/84	0.81
Infection	32/46	59/84	0.94

### Recurrence

Among 48 patients, 13 (27.1%) had recurrent LE with multiple admissions; the mean number of episodes was 2.5 (range 1–5). Duration of SLE was more in recurrent groups (7.2 ± 5.8 years’ vs 3.9 ± 2.1 years, p= 0.05). There was no statistically significant difference in clinical findings. Colon involvement was commonly seen in recurrent LE patients, though statistically insignificant. (p=0.08). Hydroureteronephrosis or cystitis was more prevalent in recurrent groups (p = 0.002) (**Table 4**). There was no statistically significant difference in mean dsDNA, C3 or C4, or SLEDAI between recurrent and nonrecurrent groups. Using the binomial logistic regression model, dsDNA, complements, and serum albumin levels failed to predict recurrence in LE (**Supplementary Table 1**).

**Table 4.** Clinical characteristics between recurrent and non-recurrent LE.

**Variables**	**Single episode (n= 35)**	**Recurrent episode (n=13)**	**p value**
Age	26.9± 8.8	27.4± 9.5	0.87
Female	33/2	12:1	-
Ascites	26	10	0.85
Hepatopathy	8	3	0.99
Pancreatitis	8	0	-
Nephritis	21	5	0.18
Serositis	27	10	0.99
Leukopenia	14	7	0.39
Arthritis	18	8	0.53
Myositis	15	0	-
Small intestine	29	9	0.30
Large intestine	23	10	0.08
Both intestines	20	5	0.25
Target sign	6	2	0.88
Hydroureteronephrosis/cystitis	6	8	0.002
No of death	8	5	0.28
Infection	9	4	0.73
Surgery	0	2	-
C3(mg/dL)	48.8± 35	40.7± 27	0.45
C4 (mg/dL)	12.6± 17	7.7± 10	0.34
dsDNA(U)	1.7± 1.6	2.1± 1.7	0.50
Albumin (g/dL)	2.3± 1.1	2.6± 0.8	0.49
APL positive	8/34	5/12	0.23
SLEDAI	14.3+8	10.5+5	0.12

### Mortality

Overall mortality was 31%. Short-term mortality (expired during enteritis admission) was 4 (9.7%), and long-term mortality (expired on a mean follow-up of 2.2 years) was 9 (21.9 %). Six (14%) patients had recurrent LE, out of which three succumbed in hospital. A logistic regression model was formulated for the prediction of mortality. The final prediction model included Age, BILAG -A domain involvement of other major organ systems, serum albumin, anti-ds-DNA, C3 and C4. A confusion matrix using Python software was performed to ensure the accuracy proportion of the right predictions (ρ) of the model. (ρ = close to 1 indicates a good model). Multiple repetitions of the confusion matrix for the mortality prediction model yielded variable ρ scores (0.5 to 0.7), suggesting that its prediction accuracy was insignificant. Individual components also failed to show statistical significance (**Supplementary Table 2**).

## DISCUSSION

LE or GI vasculitis of SLE is a potentially severe condition requiring an adequate diagnosis and urgent management.^4^ The symptoms of LE are non-specific, and most patients present with diffuse crampy or persistent abdominal pain, variable degrees of nausea and vomiting, diarrhoea, and fever.

### Clinical

The most common manifestation of LE is abdominal pain. Lian et al.^4^ reported 87% cases of abdominal pain in his series, followed by vomiting (82%). Kwok et al.^9^ revealed that among 87 cases of acute abdominal pain in lupus, 41 had the diagnosis of LE. Koo et al. reported abdominal pain, nausea, vomiting and loose motion in 93%, 65%, 54% and 46%, respectively. In our study, abdominal pain was the most common feature (89%), followed by diarrhoea (81%) and vomiting (77%). A detailed comparison of different studies and our cohort is included in **Table 5**.

**Table 5.** Comparison of different studies of LE with the present study.

**Variables**	**Chen et al.^7^**	**Lian et al.^4^**	**Kwok et al.^9^**	**Lee et al.^12^**	**Kim et al.^18^**	**Koo et al.^10^**	**Present study**
**Population**	43	52	41	17	16	46	48
**Major population**	Chinese	China	Korea	Korea		Korea	India
**Female**	38/5	98%	39/2	15/2	2/14	39/7	45/3
**Median age**	40.0 (19.0)	30	31.85 1.49	34 (12.5)	Recurrent - 30(6), non-recurrent - 32.1()	33.3 (12.7)	27.04+8.92
**Abdominal pain (%)**	88.4	87	100	100	100	93	89
**Vomiting (%)**	49	82	-	-	-	54	77
**Diarrhea (%)**	40	67	-	-	-	46	81
**SLEDAI**		6.9 +9.9	15.59 + 0.87	13.7 + 5.9	7.6–7.9	8.7 + 5.2	12.7+8.8
**Positive dsDNA**		77%	30/41	Elevated mean dsDNA	Elevated mean dsDNA	Elevated mean dsDNA	Elevated mean dsDNA
**Low complement**		53%		Low mean C3/C4	Low mean C3	Low mean C3/C4	Low mean C3
**Leukopenia**	20	4%		Fall in leukocyte count but no leukopenia	-	-	43%
**Thickened bowel**	54	63%, 30%	-	100	Mean bowel wall thickness 6–9 mm	Jejunum- 66%, Ileum −75%, Colon- 41%	Small intestine −79%, large intestine- 69%, both - 52%
**Mesenteric vessel**	74		-	-	-	19%	11/48
**Ascites**	28	56%	-	-	-	-	Serositis - 77%
**Recurrence**	1	12	13	4	4	14	13

### Disease activity

Previous studies have reported inconsistent findings regarding the association between LE and active SLE. Patients often have other evidence of active disease during an episode of LE. In the series of 52 patients by Lian et al.,^4^ majority of patients had a mean SLEDAI score of 6.9 ± 9.9, with other major organ involvement being renal (15%) and central nervous system (8%). Kwok et al.^9^ reported a mean SLEDAI of 15.59 ± 0.87. The cohort by Buck et al.^11^ reported a total of 75 admissions of subacute abdominal pain, and those patients with SLEDAI >8 developed LE. Lee et al.^12^ showed that the SLEDAI score did not differ significantly between SLE patients with abdominal pain with or without LE. Our study’s mean SLEDAI score was 12.7± 8.8, indicating that LE patients had high disease activity. However, when compared to an age- and sex-matched control population requiring admission simultaneously, the mean SLEDAI score was statistically lower in the LE group, even though dsDNA and complement levels were not statistically different. These findings again suggested that the SLEDAI score may not be suitable for assessing disease severity and making treatment decisions in patients with LE.

### Imaging

CT is the best diagnostic tool for LE. Three basic findings in CT are - bowel wall thickening, mesenteric vasodilation, and mesenteric fat attenuation. Chen et al.^7^showed 54% bowel wall thickening and 74 % mesenteric fat attenuation in their series. Byun et al.^13^ also reported a high prevalence of 79.5% (31 of 39 studies) of mesenteric vasculitis in 39 CT examinations for acute abdominal pain with symmetric bowel wall thickening in 29 cases, target sign in 26 cases and mesenteric vascular engorgement and haziness in 31 cases. In a Thai study on abdominal CT in 32 SLE patients, 24 had abdominal pain, and six (i.e. 25% of 24 patients) had CT findings consistent with LE.^14^ Ko et al.15 reported that 11 of 15 cases (73.3%) had CT features of LE. In our study, 79% of patients had small intestines, and 69% had large intestinal involvement. It can be concluded that LE is the most common cause of acute abdominal pain severe enough to necessitate imaging studies in SLE patients, both in Asia and the West. In lupus, the region supplied by the superior mesenteric artery, particularly the jejunum and ileum, is most commonly affected by vasculitis.^9,11^ However, in a study by Byun et al.,^13^ the area supplied by the inferior mesenteric artery was also found to be involved with some frequency. Gastroscopy and colonoscopy may reveal signs of ischemia and ulceration, appearing as ‘punched-out’ areas of the mucosa,^16^ also reflected in this study.

The manifestations of urinary tract damage, including hydronephrosis, ureteritis, and cystitis in LE, have been reported in the literature. Chen et al.^7^ reported that approximately 14% (6/43) of LE had hydronephrosis. In a retrospective Korean study of 1064 patients, 24 had lupus cystitis, and a fourth had a history of LE. They showed a lower C3 level (p = 0.031), higher SLEDAI (p = 0.006), and higher ESR (p = 0.05) upon admission. In our study, hydroureteronephrosis was present in 14 cases (29%), which was more common in the recurrent group (p=0.002).

### Recurrence and outcome

Koo et al.^10^ found 33 % recurrence in LE patients with a mean duration between episodes of 23.7 ± 20.2 months, and the mean number of recurrences was 2.5 (range, 1–8). In our study, 27% of patients had a recurrence, with a mean number of recurrences was 2.5 (range, 1–5). In the study above, colonic and urinary tract involvements were predictors of recurrence in LE. In our study, the colon was the most commonly involved site in the recurrent group, with higher imaging evidence of cystitis than the non-recurrent group. However, the prediction model failed to show any significant association. Kwok et al.^9^ reported that among 41 patients with LE, 13 relapsed during the study period. In relapsed cases, the median interval between two episodes was 24.4 months (range 4–131). There were no differences in demographic and clinical profiles between groups except that SLE patients with pre-existing anti-phospholipid syndrome tended to recur more frequently (p=0.028). Kim et al.^18^ reported that out of the 16 LE patients followed up for 30 months (median 67 months), nine had recurrent LE, while seven patients did not show any recurrent episodes. The two groups had no differences in demographic and laboratory indices, including autoantibody profiles and SLEDAI scores. Similarly, this study did not observe any significant difference in dsDNA, complement and SLEDAI scores between the two groups.

In most of the series, mortality was not directly attributed to LE. However, pneumonia and sepsis were identified as primary causes of death. In their case series, Koo et al.^10^ reported three deaths, all of which were attributed to pneumonia. In the study conducted by Chen et al.,^7^ it was reported that one death occurred during a follow-up period of 52 months. The cause of death was attributed to pneumocystis carinii pneumonia. Lian et al.^4^ reported three deaths in their case series occurring at 3, 38, and 52 months after their initial observation; all of them succumbed to sepsis or multiorgan failure. In our case series, four patients died during admission, and nine expired during a follow-up of 24 months.

### Risk prediction

The risk factors for LE have not been fully established. Lee et al. reported that leukopenia is significantly higher in the LE group compared to the non-LE group. Chen et al. ^7^found that a reduced C3 level and high IgA were more common in the LE group. Using logistic regression, Liu Z et al.^19^ developed a LE risk assessment (LRA) model which included C4, ANCA, albumin, anion gap, age, D-dimer, platelet, chlorine, anti-SSA, anti-Rib-P, and anti-RNP. The LRA model exhibited good consistency between predictions and actual observations in both the training and validation cohorts, suggesting the model has good prediction ability. The decision curve analysis (DCA) revealed that using the LRA model to predict LE risk in SLE patients confers a net benefit. In this study, we tried to formulate a prediction model of mortality in LE using a machine-learning algorithm; the final prediction model included age, BILAG-A domain involvement of other major organ systems, Serum albumin, Anti-dsDNA, C3 and C4. However, it failed to show any significance in mortality risk prediction. The primary limitation in this regard was a sample size with heterogeneous causes of mortality.

This study is not without its limitations. Firstly, it is crucial to acknowledge that the retrospective nature of this study may introduce bias and inaccuracies in a few records. Additionally, it should be noted that a subset of patients only had access to telephonic consultations for follow-up, potentially impacting the overall findings. The number of variables in the prediction model was reduced due to incomplete data for a few variables. The imaging report did not mention essential details, such as the thickness of the bowel wall and any improvement or changes in thickness observed during subsequent visits. The study results were also limited by not including short-term outcomes such as time to oral feeding and duration of hospital stay. Overcoming these limitations will require a further longitudinal prospective study with a pre-designed study protocol.

## CONCLUSION

The prevalence of LE in our cohort was approximately 3.2%, with a recurrence rate of 27% and a mortality rate of 31%, including both short-term and long-term mortality. Compared to the control population, individuals with LE did not show significant differences in serological activity. However, it is essential to note that SLEDAI, a global disease activity index, often misleads in LE cases as it does not include gastrointestinal manifestations. Colon and bladder involvement were more frequent in recurrent LE. Anti-dsDNA antibodies, complements, disease activity index and serum albumin failed to predict recurrence and mortality in our cohort. This study, the first of its kind in India, employed prediction analysis to gain insights into the prognosis of LE. However, a comprehensive longitudinal study incorporating more focused outcomes such as ‘the time to oral feeding’, improvement in subsequent imaging, histopathological evidence and detailed profiles of biomarkers will provide valuable insights into this specific complication of SLE.

## CONFLICT OF INTEREST

The authors declare no conflict of interest.

## FUNDING

None.

## AUTHOR CONTRIBUTIONS

Conceptualisation: SK, RB, PKD, LR; Methodology: SK, RB, YKV, AKR; Writing: RB, YKV, SK; Statistical analysis: GSR Murthy, RB, YKV, SK; Supervision: PKD, LR.

## INFORMED CONSENT

All participants gave consent for participation.

## Figures and Tables

**Supplementary Table 1.** Logistic regression showed LE association with different variables.

**LE recurrence variables**		**Score**	**df**	**Sig.**
Variables	Age	.100	1	.752
	SLE duration year	7.233	1	.007
	Gap between SLE and enteritis	2.653	1	.103
	Enteritis duration	.087	1	.768
	dsDNA	.101	1	.750
	C3	.931	1	.335
	C4	.947	1	.331
	Albumin	.366	1	.545
	SLEDAI	1.629	1	.202
Overall Statistics		14.798	9	.097

**Supplementary Table 2.** Prediction of mortality in LE using Python software (confusion matrix).

**Parameters in prediction model**	**Coefficient**	**P value**	**Int**
Age	0.02	0.718	int64
BILAG A- Constitutional	14.84	0.99	int64
BILAG A - Mucocutaneous	−38.52	0.99	int64
BILAG A - Neurological	19.48	0.99	int64
BILAG A - Musculoskeletal	−28.19	0.99	int64
BILAGA - Cardiorespiratory	63.11	1.00	int64
BILAG A - Renal	0.30	0.86	int64
BILAG A - Haematological	30.93	0.99	int64
dsDNA	17.72	0.99	float64
Serum Albumin	−19.46	0.99	float64
